# “Husband, father, coward, killer”: The discursive reproduction of racial inequality in media accounts of mass shooters

**DOI:** 10.3389/fpsyg.2022.966980

**Published:** 2022-09-29

**Authors:** Tristan Bridges, Tara Leigh Tober, Melanie Brazzell, Maya Chatterjee

**Affiliations:** ^1^Sociology Department, University of California, Santa Barbara, Santa Barbara, CA, United States; ^2^Human Development and Family Studies, Colorado State University, Fort Collins, CO, United States

**Keywords:** mass shootings, mass shooters, gun violence, perpetrators, racial inequality, media representations, media bias

## Abstract

Relying on more expansive criteria for defining “mass shootings” than much existing research, we examine a subset of a unique dataset incorporating 7,048 news documents covering 2,170 shootings in the United States between 2013 and 2019. We analyze the descriptive language used to describe incidents and perpetrators and discover significant racial disparities in representation. This research enables a critical examination of the explanatory frames utilized by news media to tell the public who mass shooters are and journalistic attempts to explain why they occur. Data were analyzed utilizing a mixed methods approach, relying on content analysis to inductively code emergent categories of descriptions of shooters and binary logistic regressions to analyze the preponderance of descriptive categories when comparing news articles reporting on shootings committed by differently racialized shooters. Our results confirm some recent research showing that mass shooters racialized as white are more likely to be described with kind and compassionate language. With our larger sample, however, we also find that mass shooters racialized as white are additionally more likely to be described with negative language as “bad” or “evil” in comparison to shooters of color. We discuss how these data demonstrate that media reports present a more complex picture of white mass shooters for the public than shooters of color.

## Introduction

An important body of scholarship has examined mass media as an important site of information about mass shootings in the United States (e.g., Schildkraut and Elsass, 2016; Murray, 2017; Dahmen, 2018; Duxbury et al., 2018; Schildkraut et al., 2018; Beard et al., 2019; Jetter and Walker, 2022). Because no truly representative national registry of mass shooting incidents exists (Smart and Schell, 2021; Bridges and Tober, 2022) and as a result of definitional discrepancies and different samples utilized by scholars of different studies (Schildkraut and Elsass, 2016; Bridges and Tober, 2022), it is difficult to answer even seemingly straightforward questions about mass shooting incidents in the United States. As a result, news media is not only an important source of information about mass shootings in the United States; for many, it may be the only source of information. As a result, how mass shootings are addressed, framed, and explained in news media reporting is consequential for the ways people make sense of and understand these violent crimes.

Some of the research examining patterns in news media coverage of mass shootings has found that perpetrators of different racial categories are given different types of coverage (e.g., Klein, 2013; McGinty et al., 2013; Fox and DeLateur, 2014; Duxbury et al., 2018). This body of work documents not only patterned differences in news media coverage by race, but argues that such coverage works in ways that shore up existing racial inequalities by relying on cultural stereotypes that justify patterns of exclusion and inequality. Generally speaking, this body of work has documented that mass shooters racialized as white tend to have their crimes treated individualistically, drawing attention to perpetrators’ mental health or unique circumstances explaining their crimes in comparison to news media accounts of shooters of color.

As Duxbury et al. (2018) explain, news media narratives attempting to explain mass shootings have changed over time. For instance, early coverage following the shooting at Columbine high school in the early 2000s used “domestic terrorism” by name in discussing incidents (e.g., Altheide, 2009). Following the domestic terrorist attack on 9/11 in the United States and the racialization of terrorism as Middle Eastern, Arab, and Muslim, however, news media narratives shifted to more individualistic explanations of mass shootings like “bullying” (e.g., Kimmel and Mahler, 2003; Leary et al., 2003; Klein, 2013). And later, news media coverage became more likely to cite mental health as an individual-level explanation for mass shootings (e.g., McGinty et al., 2013; Fox and DeLateur, 2014; Metzl and MacLeish, 2015). While some reporting does focus on the fact that the overwhelming majority of mass shootings are committed by men, less coverage considers race, despite the fact that the incidents given the most news media coverage have tended to publicize mass shootings committed by perpetrators racialized as white. Documenting this phenomenon has been an important task of social scientific research on the ways media bias has shaped what is shared with the general public about mass shootings in the United States.

Research on media coverage of crime more broadly has shown that the press often perpetuates existing beliefs about different racial groups in ways that maintain existing social hierarchies of racialized systems of inequality (i.e., Carlson, 2015). As a result, for instance, a great deal of scholarship examining racial differences of mass shootings has found that White mass shooters are more likely to be offered individual-level explanations in news media coverage of their crimes in comparison with mass shooters of color, in particular mass shooters racialized as Black. For instance, Duxbury et al. (2018) find, in a sample of 219 mass shooting incidents between 2013 and 2015, that White shooters were more likely to have their crimes attributed to mental illness, while perpetrators racialized as Black were more likely to be covered in ways that situate them as “violent threats to the public” (p. 767). This news media framing individualizes White perpetrators and offers what some scholars discuss as a “sympathetic” frame in comparison to explanations offered of mass shootings perpetrated by mass shooters of color (e.g., Metzl and MacLeish, 2015; Fox and Fridel, 2016; Duxbury et al., 2018).

Most existing scholarship has defined mass shootings in ways that produce small samples of incidents. Most research examines incidents that meet a fatality threshold of four and excludes incidents associated with gang, drug, or family and intimate partner violence (Bridges and Tober, 2022). And because the most highly publicized incidents meeting these criteria have tended to be White, some scholars have suggested that there is too little racial variation to analyze racial discrepancies in news media coverage of mass shooters and their crimes. Some, like Fox and Fridel (2016), have noted that many incidents of mass gun violence are given much less media treatment, particularly those associated with interpersonal conflicts (such as bar fights, intimate partner and family violence, and more) (see also Schildkraut and Elsass, 2016). Indeed, Fox and Fridel (2016) note that incorporating a less restrictive definition would likely increase racial diversity among incidents classified as “mass shootings.” Our data reflect this and we offer an analysis of racialized frames used to characterize mass shooters and how race is related to media coverage of mass shootings in the United States.

Scholarship has also examined the ways race plays into the ways mass shooting incidents are understood to be “newsworthy.” For instance Park et al. (2012), examined news reporting on two mass shootings that received an abundance of news coverage: the shooting at Virginia Tech and the shooting at Columbine High School. Park et al. (2012) discovered that race was more commonly mentioned in news coverage of the Virginia Tech incident (committed by a South Korean American perpetrator) than Columbine (committed by two white perpetrators). In addition to noting race as more commonly mentioned, Park et al. also discovered that media frames work to situate “the… incident around the perpetrator’s ethnicity and generalized criminal culpability to his ethnic group” (2012, 475). Similarly, in larger analyses of news coverage surrounding mass public shootings both Silva and Capellan (2019) and Fox et al. (2021) found that incidents more likely to be perpetrated by shooters racialized as white receive significantly more media attention. For instance, Fox et al. (2021) showed that shootings where victims are White, women, children, or strangers are more likely to be more widely covered as were incidents with indications of mental illness mentioned in the articles – both of which are more common for shooters racialized as white.

We build on this existing research with a much larger sample of incidents and news media stories to examine how differently racialized mass shooters are characterized in journalistic accounts of incidents. To do, so we draw upon a unique dataset we built that documents data from 2,170 shootings between 2013 and 2019 in the United States. Consistent with Booty et al.’s (2019) recommendation, we argue for a broad definition of “mass shooting,” defined by a casualty threshold of four (as opposed to a *fatality* threshold). Our definition is also not limited to assumptions about where and how mass shootings take place, including domestic and family violence as well as gang and drug violence categorically excluded from most mass shootings scholarship (e.g., Fox et al., 2019; Peterson and Densley, 2021; Follman et al., 2022). This gives us a much larger and more diverse sample than most other mass shooting databases, allowing us to more robustly analyze racial disparities in media reports of mass shootings.

Building on Duxbury et al. (2018) and others, we do find that white mass shooters were significantly more likely to be described with positive, kind, and caring language. But we also found that they were significantly more likely to be described with much more negative descriptive language, like “cold,” “twisted,” and “killer.” While this is initially counter-intuitive, we argue that this can be explained by the fact that men racialized as white who commit these crimes receive much more complex characterizations in the media when compared with shooters of color.

## Materials and methods

### Defining a “mass shooting”

Defining a “mass shooting” is itself a disputed issue (Bridges and Tober, 2022). There is not federal definition of “mass shooting,” but the closest is the Federal Bureau of Investigation’s (FBI) term “mass murder,” which consists of four or more fatalities from a single event. Many criminologists have questioned why four homicide victims, a seemingly arbitrary number, is the cutoff (Dietz, 1986; Petee et al., 1997; Holmes and Holmes, 2001; Schildkraut and Elsass, 2016; Schildkraut et al., 2018). After the highly-publicized Sandy Hook Elementary School shooting in 2012 and at the prompting of then-President Obama, Congress redefined the FBI’s criteria for defining a “mass killing” as three or more victims murdered in a single incident in a public place. This was a lower threshold than the existing definition. However, this lowered threshold does not have much scholarly justification either.

The result of this lack of definitional clarity is a lack of reliable data on mass shootings as a basis for scholarly as well as policy discussion and debate. Indeed, scholars are sometimes not talking about the same incidents when they operationalize the concept of “mass shootings.” In an analysis of discrepancies between 4 of the largest datasets relied on in academic research on the topic, Booty et al. (2019) discovered that, for the year 2017, only two incidents were included in every dataset for that year (of a total of 425 incidents from all of the databases combined). A subsequent analysis involving five of the largest databases discovered a total of 3,155 incidents between 2013 and 2020 (years for which all five databases have data) discovered that only 25 of those incidents were in all five databases (Bridges et al., 2022). We follow Booty et al.’s (2019) recommendation and agree that existing research operationalizes “mass shootings” in an arbitrarily conservative way, producing smaller samples from which we can analyze data.

Additionally, the majority of scholarship on mass shootings adopts other features of the FBI’s operationalization of “mass killings” by firearm. For instance, incidents associated with gang violence and family or intimate partner violence are categorically excluded, presumably because they are not random and do not pose a threat to the “general public.” Incidents that occur in more than a single location fail to qualify (the FBI refers to these as “spree killings”). Incidents in which there is more than a single shooter fail to qualify.

We use a more expansive definition of mass shooting which is in alignment with nonprofit private researchers the Gun Violence Archive (GVA) (Gun Violence Archive, 2022). GVA defines mass shootings as incidents where one to two shooters cause at least 4 firearm-related injuries. The Archive does not discriminate by number of fatalities and includes incidents attributed to gang violence, drug violence, family violence, and intimate partner violence. We believe the GVA focus on injury rather than fatality is sufficient to capture the “mass” traumatic impact that characterizes mass shootings in comparison to other gun violence, and that it need not discriminate by number of fatalities. Our more inclusive definition allows us to analyze a much larger sample that has traditionally been used to study mass shootings, and this larger sample enables us to answer new kinds of research questions.

### Data collection

Our research team consists of undergraduate and graduate students, who were coached and supervised in a data collection and cleaning process by Bridges and Tober. GVA gathers data on mass shootings from news sources, including local United States, national, and international news outlets. From these sources, the archive documents the date, location and corresponding Congressional districts where shootings occur, the number of injuries and fatalities, basic demographics of victims and perpetrators, and a small number of incident characteristics (for example, gang-related, shootout, domestic of family violence, etc.), and numbers of guns involved in shootings.

In this dataset, we adopt all but the latter category of data, cross-checking this information with both the news media sources GVA lists as well as additional online resources found by our research team (totaling up to six separate sources per incident). Whenever possible, we included both local and national news media outlets allowing us to verify that information we coded was documented in different news outlets. Our dataset modifies any data for which we have significant external corroboration from multiple media sources. For example, sometimes a subsequent news report will reveal that a victim of a mass shooting has died, changing them from an injury to a fatality, or that a shooting ultimately was found to have more than two shooters. This sometimes meant excluding incidents initially included in GVA’s database that do not actually meet our and GVA’s definition of a mass shooting. We also eliminate redundant cases that were sometimes counted as discrete incidents in the GVA data.^1^

We also used GVA’s sources alongside the additional news media reports we collected to code additional information not collected by GVA. Using Google News, our research team found two to six media sources for each shooting incident, prioritized by both scope and reputation of the source (only news articles originally published in English, or translated into English, are included). Because we rely on news media accounts, we cannot control for media bias and varied standards for reporting, although we are able to document those biases on a larger scale. However, using up to six varied sources when possible allows us to mitigate possible bias and confirm evidence by corroboration across multiple sources.^2^ In addition to collecting information on cases heavily reported by the media, we also document the kinds of incidents and shooters that fail to generate significant news media coverage, as we discuss in our findings.

Our research team used these articles, as well as the Gun Violence Archive data, to capture data about how a shooter was racialized by media sources. Some media sources provided a definitive racial categorization of a shooter. In other cases, our research team coded for the shooter’s “street race” (López et al., 2018), or the person’s perceived race by others in public settings, based on any available photos and/or the shooter’s name. Because this system is imperfect and subject to individual bias, our team only coded for race when we felt confident, checking in with others on the team to confirm our assessments of how perpetrators would likely be racialized by others and thus argue that that “racialization” is a better assessment of what we are measuring here than perpetrators’ racial identities.

Lastly, our research team read each news media story for an incident and hand-coded the adjectives and nouns used in the media source to describe the shooter(s) or the shooting. Following an initial analysis of descriptors used in incidents between 2013 and 2016, we inductively sorted the descriptive language into 28 separate categories *via* open coding (e.g., “humanizing,” “race,” “citizenship,” “terrorism,” “shocking,” and “physical description”) (see Table 1 and Chatterjee, 2019 for a summary).

**Table 1:** Descriptor categories for mass shooting media coverage.

Name of category	Sample of descriptors included in the category (total number of descriptors in category)
Race	For example, Russian, Native American, descent, origins (22)
Humanizing	For example, stellar, average, prominent, hardworking, remorseful, hunter, republican, retired (68)
Negative level 1	For example, possessive, cold, weird, coward, killer, twisted, isolated (35)
Negative level 2	For example, horrible, vermin, menace, brutal, ruthless, savage (23)
Uncaring	For example, heartless, indiscriminate, brazen, brutal, petty (15)
Family	For example, father, mom, son, uncle, family friend, grandson (13)
Romantic	Husband, wife, boyfriend, girlfriend (4)
Occupation	For example, employee, investor, nurse, sailor, student, mechanic (10)
Citizenship	Immigrant, alien, citizen (3)
Mass shooting	For example, mass murder, mass, massacre, mass killing (6)
Terrorism	Terrorist, extremist, terrorism (3)
Criminal record	For example, criminal record, parole, probation, prior convictions, sex offender (11)
Execution	Execution, execution-style (2)
Reckless	Reckless, brazen (2)
Shocking	For example, baffling, unbelievable, inconceivable, unreal, jaw dropping (10)
Sad	For example, tragic, heartbreaking, gut-wrenching (5)
Scary	Scary, terrifying, horrifying, horrific (4)
Gang related	Gang, gang related, gang associated, with gang ties (4)
Mental health	For example, diagnosed, schizophrenia, depression, disabilities, PTSD (16)
“Crazy”	Raving, unhinged, crazy (3)
Armed and dangerous	Armed, dangerous (2)
Domestic	Domestic, estranged, romantic (3)
Physical description	For example, height, weight, complexion, afro, buzz cut, light-skinned, tattoos (21)
Random	Random (1)
Not random	For example, not random, targeted, calculated, intentional, deliberate (6)
Drugs	For example, drug, narcotic, high, hallucinating, drug-fueled (7)
Religion	Muslim, Christian, religious, religion (4)
Other	For example, paranoid, killer, chilling, violence, murder, dispute, teen, neighbor, retaliation, kid (15)

This allowed us to examine patterns in the ways different types of shooters and shootings were portrayed in our sample. Some categories were expansive and others more specific, sometimes capturing the same descriptors twice. For instance, “Negative Level 1” includes the descriptor “cold” in addition to qualitatively different descriptors like “possessive” or “weird,” but “cold” is also included as a descriptor in a more specific category of “Uncaring” (see Table 1). This allowed us to test several levels of description specificity in our analyses. While automated text mining can provide quantitative measures of the usage of such terms, detecting more complex themes like whether the words were used to describe a shooter or just used in some other way in the articles are much more challenging and often incompletely reliable. Data were thus originally collected manually to account for the contextual relevance of the terms within the journalistic accounts of mass shootings that make up this database. These descriptive categorizations that emerged inductively are the outcome variables in our analyses in this research.

### Data analysis

Because we were interested in the influence of race on media coverage, we calculated descriptive statistics on the races of shooters in our sample (see Table 2). Nearly 60% of our sample had no racial information and it was not possible to provide racialization data for shooters. Those cases were excluded from subsequent data analysis for this manuscript. Additionally, Native American (with a total of 5 shooters), Asian (with a total of 24 shooters) and the “Other” racial category (with a total of 11 shooters) were all excluded from subsequent data analysis because they did not reach the threshold necessary for reliability. We thus used the racial categories of Black, white, and Hispanic in the regression models we present in this article. This means that we drop a large share of incidents in this analysis. But we do so because we are interested in examining racial bias in reporting on mass shooting incidents and are unable to examine this phenomenon when studying incidents in which the race of the perpetrator is not known or stated.

**Table 2:** Race of mass shooters in overall sample.

Race	Shooter 1	Percent (%)	Shooter 2	Percent (%)
Black	551	25.3	117	25.6
Hispanic	111	5.	11	2.3
White	176	8.1	7	1.5
Asian	24	1.1	2	0.4
Native American	5	0.2	1	0.2
Other	11	0.5	2	0.4
Unknown	1,297	59.6	335	70.5
Total	2,175	100.0	475	100

We also removed cases from our sample with double shooter pairs of differing races, as these interracial pairings hinder the analytic power of our research design.^3^ Descriptive statistics on the racial make-up of double shooter pairs indicates that a large majority are intra-racial pairings (White-white, Black-Black, and Hispanic-Hispanic, see Table 3). A small number of interracial pairings were excluded from the dataset for statistical insignificance (including White-Black, Hispanic-Black, White-Unknown/Asian/Native American/Other, and Hispanic-Unknown/Asian/Native American/Other). Thirty-five Black-Unknown/Asian/Native American/Other pairings were removed because their multiracial character lies outside the scope of the study’s purpose to predict the effect of a single racialized identities on media coverage. Thus, in cases of shooter pairs, the race of the first shooter is used in analysis as an index of the race of both shooters. After pruning our sample in this way, the racializations of shooters in our final sample are described in Table 4.

**Table 3:** Race of double-shooter pairs in overall sample.

Race of double-shooter pairs	Number	Percent (%)
White-white	6	3.6
Hispanic-Hispanic	10	6.0
Black-Black	108	64.6
White-Hispanic	0	0
White-Black	3	1.8
Hispanic-Black	3	1.8
White-Asian/Native American/Other/Unknown	1	0.6
Hispanic-Asian/Native American/Other/Unknown	2	1.2
Black-Asian/Native American/Other	35	20.8
Total	168	100

**Table 4:** Race of mass shooters in study’s final subsample.

Race	Shooter 1	Percent (%)	Shooter 2	Percent (%)
Black	517	65.1	106	89.1
Hispanic	105	13.2	8	6.7
White	172	21.7	5	4.2
*Total*	*794*	*100.0*	*119*	*100.0*

Knowing that we would use gender as a control variable, we also calculated the descriptive statistics of the gender of the shooters in our overall sample (Table 5), as well as the genders of double shooter pairs (Table 6). These data document a small number of women shooters (*n* = 25). By the same logic we used for removing multiracial shooter pairs, we also removed multi-gender shooter pairings (*n* = 8) to allow us to control for gender in discoveries reported in this analysis. Because gender is not our primary independent variable of concern, however, we did not remove shooters of unknown gender from the sample. The resulting sample of 794 incidents occurring between 2013 and 2019 is reported on in this manuscript (summarized in Table 7).

**Table 5:** Gender of mass shooters in overall sample.

Gender	Shooter 1	Percent (%)	Shooter 2	Percent (%)
Man	1,203	55.3	238	50.1
Woman	16	0.7	8	1.7
Unknown	956	44.0	229	48.2
Total	2,175	100	475	100

**Table 6:** Gender of double-shooter pairs in overall sample.

Gender of double-shooter pairs	Number	Percent (%)
Unknown–unknown	208	44.1
Man–unknown	21	4.4
Man–man	235	49.8
Man–woman	8	1.7
Woman–woman	0	0%
*Total*	*472*	*100.0*

**Table 7:** Gender of shooters in final subsample.

Gender	Shooter 1	Percent (%)	Shooter 2	Percent (%)
Man	774	97.5	117	98.3
Woman	11	1.4	0	0
Unknown	9	1.1	2	1.7
Total	794	100.00	119	100.0

## Results

### Index of descriptor frequency

**Figure 1:**
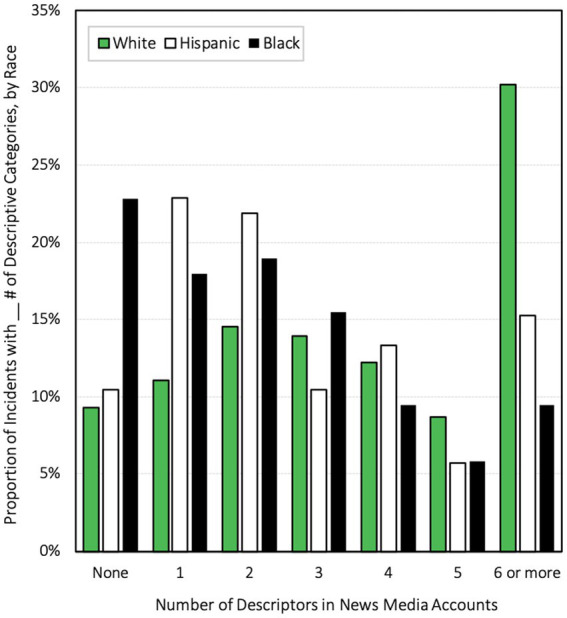
Average frequencies of descriptive categories in news media accounts of mass shootings by race, 2013-2019.

We developed an index of how many descriptions were used for each shooter based on racialization and discovered that white shooters in general received more descriptive language than Black or Hispanic shooters (see Figure 1). Noteworthy in this figure is the fact that a full 22.8% of incidents involving shooters racialized as Black received no descriptors of the shooter(s) at all – more than twice the proportion of incidents involving shooters racialized as Hispanic (10.5%) or white (9.3%). Additionally, shooters racialized as white were disproportionately overrepresented among those receiving the highest numbers of descriptions of the shooters. A full 30.2% of incidents involving shooters racialized as white included at least 6 descriptors. That is twice the proportion of incidents involving shooters racialized as Hispanic that received this descriptive diversity (14.4%) and three times the proportion of incidents involving shooters racialized as Black (9.5%).

Because we collected all of the descriptive language used to characterize shooters in media accounts and subsequently open-coded that descriptive language to identify descriptive categories, the results reported in Figure 1 are the result of more descriptive language utilized in media accounts of shootings involving perpetrators racialized as white rather than simply a larger preponderance of some particular set of descriptors for which we were looking. We discuss racial discrepancies in descriptive frequencies here because it helps us to make sense of the discoveries we report on in our regression analysis below.

### Logistic regression analysis of descriptor type usage by race

We developed a two-model logistic regression analysis to determine whether racialization could be used to predict the probabilities that a shooter was described using at least one descriptor in any of the emergent categories we identified (the dependent variables being the 28 descriptive categories we identified using the analytic strategy described previously). Model 1 (Table 8) reports on statistically significant findings among the 28 binomial logistic regressions, one for each descriptor category, using “Black” as the reference category.^4^ This model allows us to establish a baseline relationship between racialization and descriptive language in media reports that we further interrogate in Model 2 with a set of control variables.

**Table 8:** Model 1: binary logistic regression analysis of descriptor type usage in media coverage of mass shooters by race.

Descriptor by race	*χ* ^2^	*β*	SE *β*	Exp. (*β*)	95% Confidence intervals	
					Lower bound	Upper bound
Humanizing	60.397***					
White		1.587	0.204	4.888***	3.278	7.290
Hispanic		0.611	0.274	1.843*	1.077	3.154
Negative 1	24.077***					
White		0.905	0.191	2.473***	1.699	3.598
Hispanic		−0.131	0.276	0.877	0.511	1.508
Negative 2	32.293***					
White		1.083	0.191	2.953***	2.031	4.294
Hispanic		0.459	0.245	1.582*	0.978	2.560
Family	44.124***					
White		1.471	0.224	4.352***	2.805	6.752
Hispanic		0.893	0.287	2.443**	1.391	4.289
Romantic	29.179***					
White		1.685	0.365	5.392***	2.367	11.024
Hispanic		1.701	0.408	5.478***	2.461	12.195
Mass shooting	16.234***					
White		1.511	0.375	4.531***	2.171	9.458
Hispanic		0.429	0.582	1.535	0.491	4.805
Sad	43.250***					
White		1.436	0.219	4.204***	2.735	6.461
Hispanic		0.143	0.34	1.154	0.593	2.246
Gang-related	42.757***					
White		−2.839	1.015	0.058**	0.008	0.427
Hispanic		1.086	0.278	2.963***	1.717	5.112
Mental health	50.010***					
White		2.087	0.309	8.061***	4.402	14.761
Hispanic		0.886	0.443	2.426*	1.018	5.778
Domestic	26.196***					
White		1.465	0.285	4.327***	2.474	7.568
Hispanic		0.341	0.442	1.406	0.591	3.341
Observations	794					
df	2					

*p<0.10, **p<0.01, ***p<.001.

In Model 1, we find that mass shooters racialized as White are more likely than shooters racialized as Black and Hispanic to be described with a variety of positive descriptor categories, such as humanizing language; mentions of domestic, family, and romantic ties; discussions of mental health; and vocabularies of sadness. This greater likelihood on the part of shooters racialized as white is large: for these six categories of positive descriptors, statistically significant odds ratios for white shooters range from 4.204 to 8.061. Yet shootings committed by shooters racialized as white are also significantly more likely to be described using negative descriptors, too: both low level negative descriptors like “weird” or “twisted” (which we categorize as “negative 1”) in addition to more extreme negative descriptions like “savage,” “brutal,” and “ruthless” (which we categorize as “negative 2”). Descriptors for shooters racialized as Hispanic are almost never statistically significant in this analysis, meaning that descriptive categorizations do not meaningfully differ in Model one between shooters racialized as Black vs. Hispanic. One exception to this is for the descriptor “gang-related.” Here, this suggests that our findings point to not only racial bias reproduced in media coverage of mass shooting incidents, but anti-Black racial bias specifically.

In Model 2, we incorporate control variables that we were able to verify in a sample of incidents this large for the period of our analysis: state, number of victims, and whether the media coverage indicated that the shooter had a relationship to the location of the shooting. We deemed these to be variables in our dataset that might influence media coverage. We included the control variable of state because the gun and racial culture of the state where the shooting occurred might influence the portrayal of shooters by local or regional media.^5^ We included the control variable of number of victims (both injuries and fatalities) to ensure that negative descriptors like “brutal” or “cold-blooded” were not only significantly correlated with shootings with high victim counts (either injuries or fatalities). While the number of shooters in our sample of a different gender category than man is very small (11 women, 9 unknown – see Table 7), we control for gender as well to ensure that positive or negative descriptors could be accounted for by media gender bias.

Our use of a broad definition of mass shootings that includes intimate partner and family violence often excluded from other datasets certainly increases the frequency of shootings in our sample where the media may perceive domestic context as relevant. To ensure that family, romantic, and domestic descriptors were not overly accounted for because of familial and intimate partner-related gun violence, we included a control variable for domestic violence-related shootings. We code any shooting as domestic violence related if media report that it occurred between family members or romantic partners (regardless of location), or if media report that it occurred in a home (regardless of relationship between involved persons – for example, a shooting at a birthday party). We chose a broad definition in order to correct for media bias that may under-report domestic, partner, and familial violence. This was an important control as well because incidents coded as domestic violence in our dataset were also disproportionately committed by shooters racialized as white. So, we wanted to investigate whether this helped explain racialized descriptor disparities in Model 1.

Lastly, we included a control variable for shooter’s relationship to location, a variable we use as one proxy for a measurement of the perceived or reported “randomness” of a shooting. Here, we are not able to verify whether the shooter did in fact have a relationship with the location, but are able to assess whether media accounts present shooters as having a relationship with the locations in which their attacks occurred. To ensure that racialized probabilities of media coverage were not skewed by whether or not the crime was perceived to be or reported as random, we included this control. These control variables strengthen the accuracy of the second logistic regression model.

Here we report on the findings with high statistical significance from binary logistic regressions in Model 2. These confirm and strengthen some of the results of Model 1, with four descriptor categories from Model 1 retaining statistical significance in Model 2 for shooters racialized as white. Model 2 (Table 9) documents that shooters racialized as White and their shootings were 2.827 times more likely than shooters racialized as Black to be described with sad language like “tragic,” “heartbreaking,” and “gut-wrenching” than shooters racialized as Black. This confirms other work on racialized media bias presenting more sympathetic portrayals of mass shooters racialized as white.

**Table 9:** Model 2: multinomial logistic regression analysis of descriptor type usage in media coverage of mass shooters by race with controls.

Descriptor by variable	*χ* ^2^	Wald *χ*^2^	Co-efficients	Standard error	Odds ratio	95% confidence Intervals
Negative 1	102.150^***^					
Race: White		12.895^***^	0.853	0.238	2.346	1.473–3.737
Race: Hispanic		0.622	−0.264	0.335	0.768	0.398–1.481
Random		0.108	−0.067	0.205	0.935	0.626–1.397
Victims		9.042^**^	0.093	0.031	1.098	1.033–1.166
Gender: Unknown		2.529	2.115	1.330	8.290	0.612–112.340
Gender: Man		0.666	0.900	1.103	2.460	0.283–21.346
Domestic violence: Unknown		0.104	−0.182	0.563	0.834	0.277–2.514
Domestic violence: No		0.000	0	0.255	1	0.606–1.648
Family	147.338^***^					
Race: White		15.399^***^	1.096	0.279	2.992	1.731–5.173
Race: Hispanic		3.678^*^	0.712	0.371	2.038	0.985–4.218
Random		3.347^*^	−0.518	0.283	0.596	0.342–1.038
Victims		1.573	−0.053	0.043	0.948	0.872–1.031
Gender: Unknown		0.046	0.234	1.095	1.264	0.148–10.816
Gender: Man		1.060^**^	−2.106	0.792	0.122	0.026–0.576
Domestic violence: Unknown		2.121	−0.882	0.606	0.414	0.126–1.357
Domestic violence: No		24.770^***^	−1.355	0.272	0.258	0.151–0.440
Sad	154.981^***^					
Race: White		13.378^***^	1.039	0.284	2.827	1.620–4.933
Race: Hispanic		0.109	0.137	0.414	1.147	0.509–2.584
Random		0.011	−0.029	0.278	0.972	0.564–1.675
Victims		20.248^***^	0.191	0.043	1.211	1.114–1.316
Gender: Unknown		389.566^***^	16.28	0.825	11,758,249	2334768.657–59216324.9
Gender: Man		.	14.549	0	1082988.588	2082988.588–2082988.588
Domestic violence: Unknown		3.538^*^	−1.621	0.862	0.198	0.037–1.070
Domestic violence: No		16.120^***^	−1.188	0.296	0.305	0.171–0.544
Mental Health	121.937^***^					
Race: White		18.442^***^	1.619	0.377	5.048	2.411–10.569
Race: Hispanic		1.982	0.899	0.521	2.457	0.886–6.818
Random		6.915^**^	−1.115	0.424	0.328	0.143–0.753
Victims		6.424^*^	0.074	0.029	1.077	1.017–1.140
Gender: Unknown		0.011	9.286	88.999	10785.026	1.892E−72–6.147E+79
Gender: Man		0.010	8.680	88.990	5885.118	1.050E−72–3.299E+79
Domestic violence: Unknown		0.058	−0.267	1.108	0.766	0.087–6.719
Domestic violence: No		0.547	−0.267	0.361	0.766	0.377–1.553
Observations	794					
df	54	1				

*p < 0.10; **p < 0.01; ***p < 0.001.

State is also included as a control variable in the analyses in this table, but not listed here for space. This control did not impact the relationships analyzed here.

Despite controlling for domestic violence-related shootings, Model 2 shows that media coverage of shootings committed by perpetrators racialized as white (and to a less statistically significant extent also incidents committed by perpetrators racialized as Hispanic) more often employ descriptors related to family. For example, shooters racialized as white were 2.992 times more likely to be described using familial terms (son, father, uncle, mom, etc.) than shooters racialized as Black, controlling for domestic violence-related incidents. This descriptive language serves to contextualize and humanize white but not Black shooters as part of larger family and community systems.

Our findings also confirm those of Metzl and MacLeish (2015) and Duxbury et al. (2018) that mass shootings committed by white perpetrators are more likely to be covered in the media using a mental health framework. Among all the descriptors, mental health shows the greatest differences between shooters racialized as white and those racialized as Black: white shooters were 5.048 times more likely to have their mental health mentioned in media sources than shooters racialized as Black. Perpetrators racialized as Hispanic were also 2.457 times more likely to be described with mental health language such as “diagnosed,” “depression,” “disabilities,” and “PTSD” [post-traumatic stress disorder]. The finding for shooters racialized as Hispanic here was less statistically significant (*p* < 0.1).

On the other hand, shooters racialized as white also continued to receive more negative descriptors in media coverage. Shooters racialized as white and their shootings were 2.346 times more likely to be described with negative terms on the milder end of the spectrum (such as “cold,” “weird,” “coward,” “twisted”) than shooters racialized as Black and their shootings. In this regard, Model 2 also strengthens and confirms Model 1’s unconventional finding that White shooters/shootings receive both more positive *and* more negative descriptors than other racial groups in the analysis. But once controlling for domestic violence-related incidents, shooters racialized as white were no longer found to also receive the most negative descriptor category in the study including language like “savage,” “vermin,” “horrible,” and “brutal.” The preponderance of mass shooters racialized as white among those committing crimes we coded as domestic violence accounted for that more negative descriptive language.

### Summary of findings

Both models in our analysis found that both negative and positive descriptive language were significantly more common in articles reporting on mass shootings involving shooters racialized as white when compared with those racialized as Black. At first, this might strike some as counterintuitive and challenges some of the conventional wisdom surrounding racial biases in news media reporting. We suggest, however, that a plausible and likely explanation for this discovery relates to the fact that mass shooters racialized as white simply receive *more* descriptive diversity in media coverage of their crimes. The frequencies of incidents involving different numbers of descriptors that we present in Figure 1 is useful to consult here.

These data suggest that mass shooters racialized as white in the media do in fact receive a variety of more positive and humanizing language (e.g., “neighbor,” “hardworking,” “nice,” “kind,” “warm,” “dedicated,” “articulate,” “responsible,” “cooperative,” and “likable”). These are strange descriptions to read in articles reporting on perpetrators of extreme gun violence. But they play a role in presenting incidents involving shooters racialized as white as surprising or unexpected when compared with media accounts of shootings committed by shooters racialized as Black or Hispanic. They are also part of the more sympathetic framing existing scholarship has documented as more common in media reporting on mass shootings involving perpetrators racialized as white. Conversely, the different media treatment associated with shootings committed by Black and Brown perpetrators is implicitly situated as more “expected” when compared to descriptive treatments afforded shooters racialized as white.

Counterintuitively, this descriptive inequity also involved more negative descriptions of shooters racialized as white when compared with shooters racialized as Black and Hispanic in our study. And this is a relatively novel finding in research on mass shootings. Because more descriptive language is dedicated to incidents involving mass shooters racialized as white, however, it is not necessarily surprising that they are also overrepresented here. And because mass shooters racialized as white in our sample receive so much more descriptive diversity than do shooters racialized as Black or Hispanic, media portrayals and descriptions of those incidents and perpetrators are simply much more nuanced and complex. Indeed, alongside the abundance of descriptive language accompanying media accounts of mass shooters racialized as white, we also discovered incidents involving perpetrators racialized as Black and Hispanic to be significantly more likely to have a complete absence of descriptive language in new media reporting. This descriptive absence is particularly over-represented among incidents involving shooters racialized as Black (see Figure 1).

## Discussion

This study reports on the largest database of news media accounts of mass shootings since 2013 of which we are aware, examining categories of descriptive language relied upon in media reports for shooters racialized in different ways. Our analysis demonstrates that mass shooters racialized as white in media accounts are more likely to be described and characterized in media accounts of their shootings using positive, kind, and caring frames. We also found, however, that mass shooters racialized as white are also significantly more likely to be framed with negative, bad, and evil descriptive language, complicating an easy summary of what this means. We suggest that these twin findings support the fact that white mass shooters simply receive much more complex descriptive characterizations in the news media in comparison to shooters of color. Building on Duxbury et al. (2018), Silva and Capellan (2019), Fox et al. (2021), and others, this adds to our understandings of how mass shootings are deemed newsworthy adding new findings explain how the racialization of perpetrators illustrates that race plays a critical role in this process and shapes what is shared about mass shooters and their crimes.

This matters for a collection of reasons. Among them, it matters because it contributes new data and analyses to the discovery that mass shooters racialized as white receive more individualistic treatment by news media in comparison with mass shooters of color. It also supports prior explanations of media accounts of mass shootings committed by shooters racialized as white that have documented a larger reliance on individualistic or psychological explanations in comparison with research on shootings committed by men of color, which are more likely to receive structural or cultural explanations that operate in ways that implicitly (and sometimes explicitly) shore up racist understandings of gun violence in the United States (Metzl, 2019). Similarly, similar to Duxbury et al. (2018), we also discovered that perpetrators racialized as white were significantly more likely to be offered descriptors that present a sympathetic frame. The racial bias we document here, however, is best understood as anti-Black racial bias as mass shooters racialized as Black receive the least descriptive language of any group. We suggest that this deficit of descriptive language works collectively to implicitly frame Black racial and ethnic groups as having more of a generalized criminal culpability, consistent with Park et al.’s (2012) analysis of coverage of the incident at Virginia Tech in comparison to Columbine High School.

Media accounts encourage the public to make sense of mass gun violence in particular ways. And the racial descriptive discrepancies we document in this study suggests that the public are being encouraged to make sense of incidents involving shooters racialized as white in very different, more nuanced and complex ways in comparison to incidents committed by men of color, and in particular in comparison to perpetrators racialized as Black. Further research is needed on this topic, and research incorporating less restrictive definitions of incidents, allowing us to look at larger samples of incidents that ought to be in more dialogue with research on mass shootings in the United States.

## Data availability statement

The raw data supporting the conclusions of this article will be made available by the authors, without undue reservation.

## Author contributions

All authors listed have made a substantial, direct, and intellectual contribution to the work and approved it for publication.

## Funding

This research was supported by a grant from the Pahl Initiative for the Study for Critical Social Issues at University of California, Santa Barbara.

## Conflict of interest

The authors declare that the research was conducted in the absence of any commercial or financial relationships that could be construed as a potential conflict of interest.

## Publisher’s note

All claims expressed in this article are solely those of the authors and do not necessarily represent those of their affiliated organizations, or those of the publisher, the editors and the reviewers. Any product that may be evaluated in this article, or claim that may be made by its manufacturer, is not guaranteed or endorsed by the publisher.

## Footnotes

^1^For the period in consideration in this article (2013–2019), the GVA database includes 2,950 incidents. After our cleaning of the dataset, we include 2,170 incidents in the period analyzed here, meaning that 780 were excluded from analysis because the incidents do not meet the criteria for inclusion as defined by GVA.

^2^We limited news sources to six in part for feasibility reasons as news media articles were all hand-coded by our research team. We are also in the process of conducting a textual analysis of the archive of news media articles in our database. And because some incidents generate so much more news media than others, that larger analysis would have exaggerated findings from incidents that happened to receive far greater news media attention in comparison with other incidents. For these reasons, we limited the number of articles to six per incident with a system for attempting to gather articles from the most reputable and influential news sources reporting on each incident.

^3^A substantial number of incidents in the GVA database are shooting reports of incidents where little is known about the incident other than the numbers of fatalities and injuries. While we do not address this in this manuscript, in other work we are examining patterns in incidents about which we have very little information (like the sociodemographic makeup of the communities in which those incidents occur). Indeed, previous scholarship suggests that shooting incidents where the victims and perpetrators are men of color, that do not happen in schools or workplaces, and where mental health is not mentioned receive substantially and significantly less news coverage (e.g., Silva and Capellan, 2019; Fox et al., 2021). This suggests that the findings in this article might be even more exaggerated if a large share of incidents we cannot analyze here were in fact committed by shooters of color, Black shooters in particular. Here these incidents are excluded because we cannot analyze potential racial bias in media reporting on incidents for which the racialization(s) of the shooters is/are not known.

^4^We rely on Black as the reference category here for two reasons. One is that models using culturally dominant groups as the reference categories sometimes operate in ways that implicitly frame culturally dominant groups as "normal" - statistically examining how various Others vary from what is framed as the norm (e.g., Johfre and Freese, 2021). Here, we select Black as the underabundance of descriptors used in news articles on Black shooters helps us to better appreciate the media treatments groups racialized in other ways receive in comparison to Black shooters.

^5^For more on state-level measures of gun culture, see Boine et al., 2020.
